# Risk of Second Non-Breast Primary Cancer in Male and Female Breast Cancer Patients: A Population-Based Cohort Study

**DOI:** 10.1371/journal.pone.0148597

**Published:** 2016-02-19

**Authors:** Man-Hsin Hung, Chia-Jen Liu, Chung-Jen Teng, Yu-Wen Hu, Chiu-Mei Yeh, San-Chi Chen, Sheng-Hsuan Chien, Yi-Ping Hung, Cheng-Che Shen, Tzeng-Ji Chen, Cheng-Hwai Tzeng, Chun-Yu Liu

**Affiliations:** 1 Department of Oncology, Taipei Veterans General Hospital, Taipei, Taiwan; 2 Division of Hematology and Oncology Department of Medicine, Taipei Veterans General Hospital, Taipei, Taiwan; 3 Department of Family Medicine, Taipei Veterans General Hospital, Taipei, Taiwan; 4 School of Medicine, National Yang-Ming University, Taipei, Taiwan; 5 Program in Molecular Medicine, School of Life Sciences, National Yang-Ming University and Academia Sinica, Taipei, Taiwan; 6 Institute of Public Health, National Yang-Ming University, Taipei, Taiwan; 7 Institute of Biopharmaceutical Sciences, National Yang-Ming University, Taipei, Taiwan; 8 Division of Oncology and Hematology, Department of Medicine, Far Eastern Memorial Hospital, Taipei, Taiwan; 9 Department of Psychiatry, Chiayi Branch, Taichung Veterans General Hospital, Chiayi, Taiwan; 10 Department of Information Management, National Chung-Cheng University, Chiayi, Taiwan; ENEA, ITALY

## Abstract

Female breast cancer patients have an increased risk of developing subsequent malignant diseases, but this issue is rarely discussed in regards to male breast cancer patients. Thus, we conducted a national survey that included 100,915 female and 578 male breast cancer patients to investigate the risk of second primary malignancy (SPM). During a follow-up period that included 529,782 person-years, 3,153 cases of SPM developed. Compared with the general population, the standardized incidence ratio (SIR) of SPM in breast cancer patients was 1.51 [95% confidence interval (CI): 1.46–1.56]. The observed risk was significantly higher in male patients (SIR 2.17, 95% CI 1.70–2.73) and in patients whose age at breast cancer diagnosis was 40 years or younger (SIR 3.39, 95% CI 2.80–4.07), comparing to age-matched general population. Compared with the overall female population, the SIRs of female breast cancer patients with uterine (SIR: 2.66, 95% CI: 2.37–2.98), thyroid (SIR: 2.30, 95% CI: 2.02–2.62), and bone and soft tissue (SIR: 2.16, 95% CI: 1.56–2.91) cancers were significantly increased. Male breast cancer patients also displayed significantly higher SIRs for thyroid (SIR: 13.2, 95% CI: 1.60–47.69), skin (SIR: 8.24, 95% CI: 3.02–17.94) and head and neck (SIR: 4.41, 95% CI: 2.35–7.54) cancers. Among breast cancer patients, risk factors significantly associated with SPM included male gender, older age, chemotherapy treatment and comorbidity with liver cirrhosis. From our analysis, we concluded that the risk of SPM was significantly higher for both male and female breast cancer patients compared with the general population, suggesting that more intensive surveillance may be needed, especially in high-risk patients.

## Introduction

Breast cancer is one of the most common cancers worldwide and accounts for nearly a quarter of the newly diagnosed cancer cases reported for female patients each year.[1,2] Although rare, increased incidence in male breast cancer has been observed.[3–5] The overall survival of breast cancer patients, both male and female, has greatly improved in recent years.[6] The higher incidence of breast cancer and favorable disease outcome make issues for long-term survivors, especially the development of another malignant disease after breast cancer, become a more pertinent consideration.[7,8]

The risk of a second primary malignancy (SPM) in breast cancer patients has previously been discussed.[7–11] In general, the overall risk for developing a non-breast primary malignancy in female breast cancer patients has been reported to range from 18% to 30%, with higher incidences associated with early onset breast cancer diagnosis.[7–9] Using the Taiwan National Cancer Registry, Lee et al. demonstrated that female breast cancer patients, particularly those diagnosed with breast cancer at 50 years of age or younger, were associated with an increased SPM risk ratio.[8] However, these observations were restricted to female breast cancer patients, and there was no further discussion regarding the possible influence of treatments. Therefore, to better understand the risk of SPM in Asian male breast cancer patients, as well as the possible effects that treatment may have on both genders, a comprehensive investigation is needed.

Taiwan’s National Health Insurance (NHI) program provides comprehensive coverage to over 23 million people in Taiwan and is a valuable resource for investigating malignant diseases in breast cancer patients.[12] The nationwide population-based study conducted by the National Health Insurance Research Database (NHIRD) was analyzed in order to clarify the incidence rate, site of involvement, potential risk factors and subsequent outcome of second non-breast primary malignancies that affected breast cancer patients, including males.

## Materials and Methods

### Data Sources

This study was approved by the ethics committee of the Institutional Review Board of Taipei Veterans’ General Hospital. The NHI program was started in 1995 as a mandatory universal health insurance program in Taiwan. Currently, this program provides comprehensive medical care for more than 99% of Taiwanese residents.[12] Extensive coverage of various medical services is provided, including outpatient, inpatient, emergency, dental, traditional Chinese medicine services, and prescription drugs. The registration database for the NHI program is managed by the National Health Research Institute of Taiwan and was used in this study. Specifically, the NHI Database of Catastrophic Illness provides comprehensive enrollment information for all patients diagnosed with severe diseases, including cancer. All malignancies are considered catastrophic illnesses, and the certification of any malignancy requires tissue pathologic proof for peer review [13]. Confidentiality is maintained according to data regulations of the Bureau of National Health Insurance and the NHRI. In line with these guidelines, the present study was exempt from a full review by the institutional review board because the NHI dataset consists of de-identified secondary data for research purposes.

### Study Population and Diagnosis of Breast Cancer

Patients with breast cancer were retrieved from the Registry of Catastrophic Illness according to the International Classification of Diseases, Ninth Revision, Clinical Modification (ICD-9-CM) code 174–175. Cases registered between the dates of January 1, 1997, and December 31, 2011, were analyzed. In Taiwan, the application for a certificate of cancer-associated catastrophic illness requires pathological proof of malignancy with corresponding laboratory findings and imaging examinations provided for peer review. To avoid any bias, the following patient groups were excluded: patients diagnosed with breast cancer before January 1, 1997, patients diagnosed with malignancies prior to their breast cancer diagnosis (n = 2341), patients who completed less than one year of follow-up following a breast cancer diagnosis (that is, those patient whose breast cancer diagnosis were after December 31, 2010) (n = 95) and three patients who lacked complete personal information in the database. The exclusion of patients registered between 1995 to the date of January 1, 1997 is to avoid any patients whose breast cancers were diagnosed before the start of NHI but later registered in database as newly-diagnosed breast cancer. In addition, information was relatively more robust after Jan 1, 1997. Information on comorbidities, including diabetes mellitus (DM), chronic obstructive pulmonary disease (COPD), chronic kidney disease (CKD), dyslipidemia, liver cirrhosis and tuberculosis were also collected from the NHIRD.

### Statistical Analysis

The main dependent variable analyzed was the occurrence of a second primary cancer. Subjects with another pathologically confirmed cancer diagnosis were identified from the Registry for Catastrophic Illness. Patients from the breast cancer cohort were followed until the development of cancer, death, dropout from the NHI program or until December 31, 2011.

The risk of a second primary cancer among the breast cancer cohort was determined using standardized incidence ratios (SIRs). This ratio includes the observed versus expected number of cancer occurrences. The expected number of cancers was calculated by multiplying the national incidence rate of various cancers according to gender, patient age (in 5-year intervals) and calendar year by the corresponding stratum-specific person-time accrued in the cohort.[12] The incidence rates for cancers among the general population were obtained from the Taiwan National Cancer Registry. For the SIRs, 95% confidence intervals (CIs) were estimated by assuming that the observed number of cancers followed a Poisson probability distribution. SIRs were also determined for patient subgroups according to gender and age. To avoid potential surveillance bias, subgroup analysis was stratified according to the time since a breast cancer diagnosis. SIRs were also estimated for each cancer type.

Demographic data, including patient age, gender, and comorbidities were compared for patients with and without second non-breast primary cancers using Fisher’s exact test for categorical variables and the Mann-Whitney U test for continuous variables. Univariate and multivariate Cox proportional hazard models were also utilized to identify predictors of second non-breast primary cancer development among patients previously diagnosed with breast cancer.

Datasets were extracted and computed using the Perl programming language (version 5.12.2). A Microsoft SQL Server 2008 (Microsoft Corp., Redmond, WA, USA) was used for data linkage, processing, and sampling. All statistical analyses were performed using SPSS statistical software version 19.0 for Windows (SPSS, Inc., Chicago, IL, USA). A p-value less than 0.05 was considered statistically significant.

## Results

### Study population characteristics

There were 101,493 breast cancer patients identified from the Catastrophic Illness Registry of the NHIRD. Of these patients, 100,915 (99.43%) were female and 578 (0.57%) were male. Overall, this cohort was observed for 529,782 person-years from January 1997 to December 2011, with a median follow-up period of 4.28 years [interquartile range (IQR): 1.86–7.96]. As shown in Table 1, male patients tended to be older, have more comorbidities and received less chemotherapy and radiotherapy than female patients. Demographic data and treatment details for both gender groups are shown in Table 1.

**Table 1:** Characteristics of patients with breast cancer.

	Total	Male	Female
Number of patients	101,493	578	100,915
Person-years at risk	529,782	2,772.73	527,009
Median follow-up, years (IQR)	4.28(1.86–7.96)	3.85(1.60–7.15)	4.29(1.87–7.96)
Median age, years (IQR)	50(44–59)	66(54–76)	50(44–59)
Age at diagnosis, years			
20–29	1484 (1.5%)	3 (0.5%)	1481 (1.5%)
30–39	12133 (12.0%)	28 (4.8%)	12105 (12.0%)
40–49	33995 (33.5%)	67 (11.6%)	33928 (33.6%)
50–59	28572 (28.2%)	119 (20.6%)	28453 (28.2%)
60–69	15491 (15.3%)	129 (22.3%)	15362 (15.2%)
70–79	7387 (7.3%)	150 (26.0%)	7237 (7.2%)
≥ 80	2431 (2.4%)	82 (14.2%)	2349 (2.3%)
**Comorbidities**			
Diabetes mellitus	18,505 (18.2%)	132 (22.8%)	18,373 (18.2)
COPD	15,809 (15.6%)	149 (25.8%)	15,660 (15.5)
Chronic kidney disease	8,264 (8.1%)	69 (11.9%)	8,195 (8.1)
Liver cirrhosis	1,296 (1.3%)	20 (3.5%)	1,276 (1.3)
Autoimmune diseases	8,227 (8.1%)	31 (5.4%)	8,196 (8.1)
Dyslipidemia	23,801 (23.5%)	134 (23.2%)	23,667 (23.5)
**Breast Cancer Treatment**			
**Hormone treatment**	71,982 (70.9%)	383 (66.3%)	71,599 (70.9%)
Anastrozole	13,174 (13.0%)	37 (6.4%)	13,137 (13%)
Letrozole	13,883 (13.7%)	38 (6.6%)	13,845 (13.7%)
Exemestane	6,867 (6.8%)	16 (2.8%)	6,851 (6.8%)
Tamoxifen	66,950 (66.0%)	367 (63.5%)	66,583 (66%)
**Chemotherapy**	71,842 (70.8%)	273 (47.2%)	71,569 (70.9%)
Anthracycline	53,672 (52.9%)	165 (28.5%)	53,507 (53%)
Taxane	25,837 (25.5%)	83 (14.4%)	25,754 (25.5%)
Cyclophosphamide	65,270 (64.3%)	198 (34.3%)	65,072 (64.5%)
Fluoropyrimidine	60,064 (59.2%)	221 (38.2%)	59,843 (59.3%)
Methotrexate	15,039 (14.8%)	47 (8.1%)	14,992 (14.9%)
Vinorelbine	9,215 (9.1%)	30 (5.2%)	9,185 (9.1%)
Platinum	8,059 (7.9%)	40 (6.9%)	8,019 (7.9%)
Trastuzumab	7,064 (7.0%)	13 (2.2%)	7,051 (7.0%)
**Radiotherapy**	46,581 (45.9%)	162 (28%)	46,419 (46%)

### Second primary malignancy occurrence after breast cancer diagnosis

**Fig 1:**
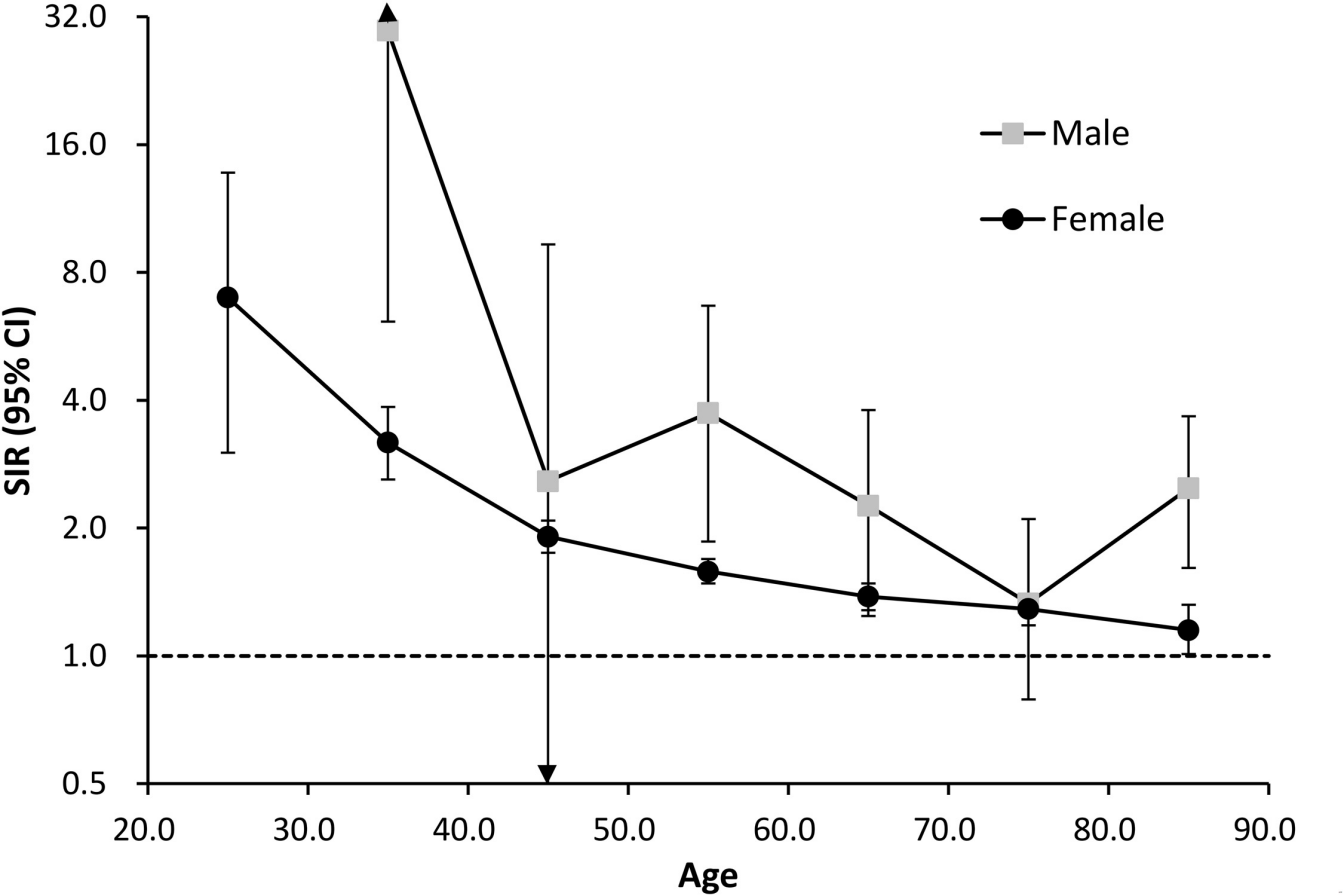
The standard incidence ratios (SIRs) of second primary malignancy in male and female breast patients are influenced by the age of breast cancer diagnosis. Point: SIR, bar: 95% confidence interval.

During the observation period, 3,153 breast cancer patients developed subsequent malignant disease. The SIR for a second non-breast primary cancer in these patients was 1.51 [95% CI: 1.46–1.56]. Further subset analysis demonstrated that the calculated SIR was higher for male patients (SIR: 2.17; 95% CI: 1.70–2.73) compared with female patients (SIR: 1.50; 95% CI: 1.44–1.55). Furthermore, a significant increase in SIR of second primary cancer was observed in all patients whose breast cancer was diagnosed at an age younger than 40 years (SIR: 3.39, 95% CI 2.80–4.07), but this risk decreased as the patient age at the time of breast cancer diagnosis increased (Table 2 and Fig 1). It is important to note that the SIRs for male patients in all of the different age subgroups were higher than those observed for female patients (Fig 1). A summary of the sub-analyses performed is provided in Table 2.

**Table 2:** Standardized incidence ratios according to gender, age at diagnosis and duration of breast cancer.

	Total			Male			Female		
Characteristics	Observed	Expected	SIR (95% CI)	Observed	Expected	SIR (95% CI)	Observed	Expected	SIR (95% CI)
All cancers	3,153	2,090.51	1.51(1.46–1.56)	73	33.63	2.17(1.70–2.73)	3,080	2,056.89	1.50(1.44–1.55)
Age at diagnosis, years									
20–29	8	1.15	6.96(3.01–13.72)	0	0.00	0.00(0.00–1518.06)	8	1.15	6.98(3.01–13.75)
30–39	106	32.47	3.26(2.67–3.95)	3	0.10	29.70(6.12–86.80)	103	32.37	3.18(2.60–3.86)
40–49	510	266.60	1.91(1.75–2.09)	2	0.78	2.58(0.31–9.31)	508	265.82	1.91(1.75–2.08)
50–59	928	582.02	1.59(1.49–1.70)	11	2.95	3.73(1.86–6.68)	917	579.07	1.58(1.48–1.69)
60–69	774	556.36	1.39(1.29–1.49)	14	6.19	2.26(1.24–3.79)	760	550.17	1.38(1.28–1.48)
70–79	582	451.33	1.29(1.19–1.40)	18	13.55	1.33(0.79–2.10)	564	437.78	1.29(1.18–1.40)
≥ 80	245	200.59	1.22(1.07–1.38)	25	10.07	2.48(1.61–3.67)	220	190.53	1.15(1.01–1.32)
Duration of breast cancer									
0–1	479	328.54	1.46(1.33–1.59)	15	5.64	2.66(1.49–4.38)	464	322.90	1.44(1.31–1.57)
1–5	1,456	957.61	1.52(1.44–1.60)	37	16.00	2.31(1.63–3.19)	1,419	941.61	1.51(1.43–1.59)
≥ 5	1,218	804.37	1.51(1.43–1.60)	21	11.99	1.75(1.08–2.68)	1197	792.38	1.51(1.43–1.60)

SIR Standardized incidence ratio; CI confidence interval; N/A not applicable.

### The SIRs for Specific Cancers

**Fig 2:**
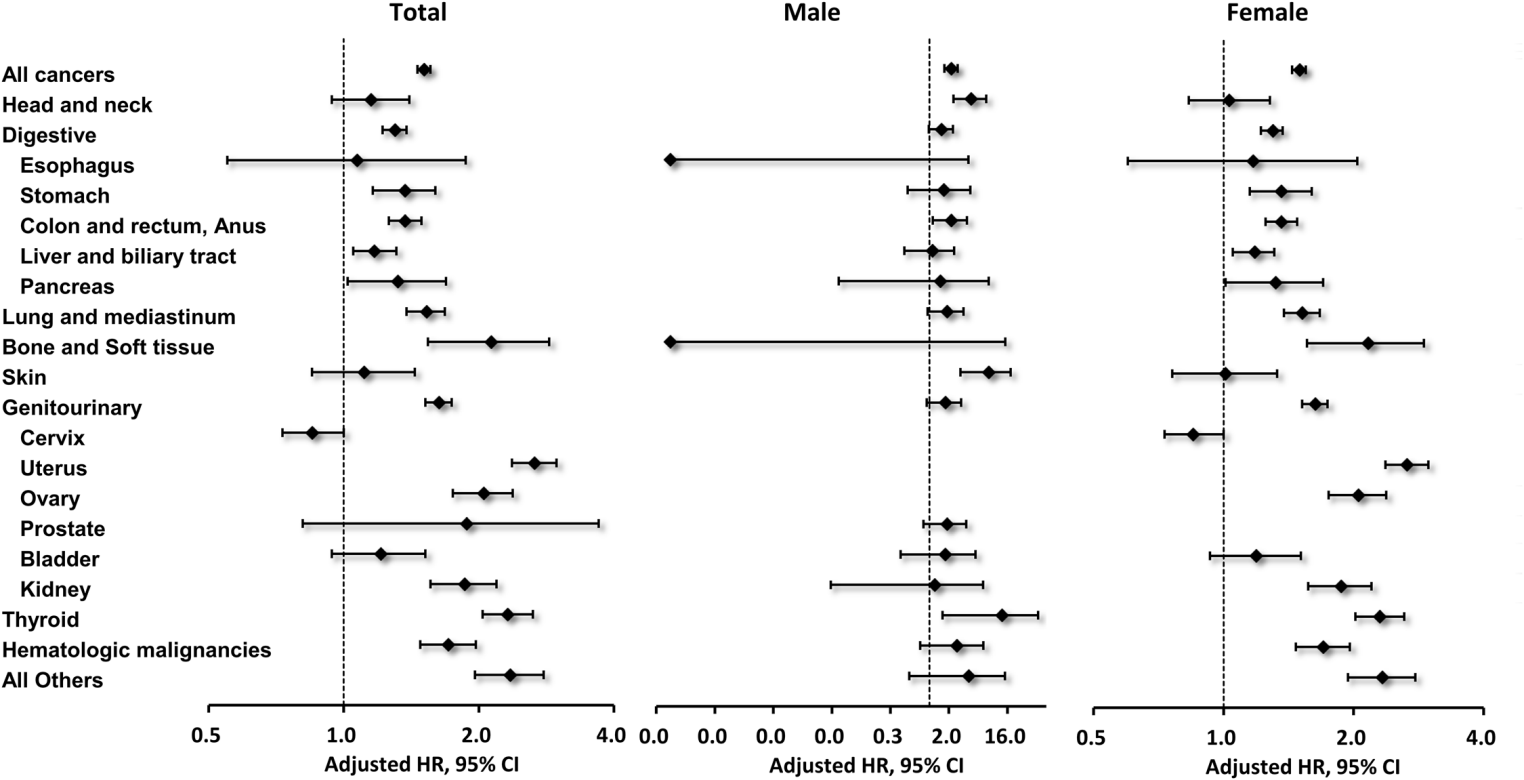
The standard incidence ratios (SIRs) of specific type of second non-breast primary malignancy in male and female breast patients. Point: SIR, bar: 95% confidence interval.

As shown in Fig 2, the SIRs for specific types of second primary malignancies in breast cancer patients were found to vary according to gender. For example, in female patients, the most commonly observed cancer types were colon and rectal (n = 543), followed by lung and mediastinum (n = 415) and uterus (n = 298) (Table 3) cancers. Relative to the general population, female patients with a prior breast cancer diagnosis were found to have a significantly higher risk of developing a subsequent cancer of the uterus (SIR: 2.66; 95% CI: 2.37–2.98), thyroid (SIR: 2.30; 95% CI: 2.02–2.62), ovary (SIR: 2.05; 95% CI: 1.75–2.38), kidney (SIR: 1.87; 95% CI: 1.57–2.20), stomach (SIR: 1.36; 95% CI: 1.15–1.60), liver (SIR: 1.18; 95% CI: 1.05–1.31), pancreas (SIR: 1.32; 95% CI: 1.01–1.70), lung and mediastinum (SIR: 1.52; 95% CI: 1.38–1.67), bone and soft tissue (SIR: 2.16; 95% CI: 1.56–2.91), as well as hematologic malignancies (SIR: 1.70; 95% CI: 1.47–1.96). For male patients, head and neck cancer was the most common second primary malignancy after breast cancer (n = 13), followed by colon and rectum (n = 12) and lung and mediastinum (n = 11) cancers. However, compared with the general population, the risk of thyroid cancer (SIR: 13.2; 95% CI: 1.60–47.69), skin cancer (SIR: 8.24; 95% CI: 3.02–17.94), colon cancer (SIR: 2.18; 95% CI: 1.31–3.80), and head and neck cancer (SIR: 4.41; 95% CI: 2.35–7.54) were markedly higher in male patients. To investigate the influence of a potential surveillance bias, SIR analyses were also performed, which excluded patients with second primary cancers diagnosed within one year after the initial breast cancer diagnosis. This model yielded similar results (Table 3 and Table A in S1 File).

**Table 3:** Standardized incidence ratios for specific cancer types among patients with breast cancer (follow-up more than 1 year).

	Total			Male			Female		
Site of cancers	Observed	Expected	SIR (95% CI)	Observed	Expected	SIR (95% CI)	Observed	Expected	SIR (95% CI)
All cancers	2,674	1,761.98	1.52(1.46–1.58)	58	27.99	2.07(1.57–2.68)	2,616	1,733.99	1.51(1.45–1.57)
Head and neck	91	71.47	1.27(1.03–1.56)	10	2.41	4.15(1.99–7.63)	81	69.06	1.17(0.93–1.46)
Digestive	978	732.09	1.34(1.25–1.42)	21	12.37	1.70(1.05–2.60)	957	719.73	1.33(1.25–1.42)
Esophagus	10	9.53	1.05(0.50–1.93)	0	0.76	0.00(0.00–4.85)	10	8.77	1.14(0.55–2.10)
Stomach	134	92.63	1.45(1.21–1.71)	4	1.98	2.02(0.55–5.18)	130	90.65	1.43(1.20–1.70)
Colon and rectum, Anus	484	342.67	1.41(1.29–1.54)	10	4.60	2.18(1.04–4.00)	474	338.07	1.40(1.28–1.53)
Liver and biliary tract	291	246.66	1.18(1.05–1.32)	6	4.46	1.34(0.49–2.93)	285	242.20	1.18(1.04–1.32)
Pancreas	59	40.60	1.45(1.11–1.87)	1	0.56	1.77(0.04–9.87)	58	40.04	1.45(1.10–1.87)
Lung and mediastinum	352	238.05	1.48(1.33–1.64)	10	4.88	2.05(0.98–3.77)	342	233.17	1.47(1.32–1.63)
Bone and Soft tissue	38	16.86	2.25(1.60–3.09)	0	0.21	0.00(0.00–17.78)	38	16.65	2.28(1.62–3.13)
Skin	44	44.55	0.99(0.72–1.33)	3	0.61	4.88(1.01–14.26)	41	43.94	0.93(0.67–1.27)
Genitourinary	719	433.70	1.66(1.54–1.78)	9	5.72	1.57(0.72–2.99)	710	427.99	1.66(1.54–1.79)
Cervix	131	150.04	0.87(0.73–1.04)	N/A	N/A	N/A	131	150.04	0.87(0.73–1.04)
Uterus	265	94.71	2.80(2.47–3.16)	N/A	N/A	N/A	265	94.71	2.80(2.47–3.16)
Ovary	142	69.81	2.03(1.71–2.40)	N/A	N/A	N/A	142	69.81	2.03(1.71–2.40)
Prostate	7	3.61	1.94(0.78–4.00)	7	3.61	1.94(0.78–4.00)	N/A	N/A	N/A
Bladder	60	50.83	1.18(0.90–1.52)	2	1.42	1.41(0.17–5.09)	58	49.41	1.17(0.89–1.52)
Kidney	114	64.70	1.76(1.45–2.12)	0	0.69	0.00(0.00–5.36)	114	64.02	1.78(1.47–2.14)
Thyroid	183	85.21	2.15(1.85–2.48)	1	0.12	8.06(0.20–44.93)	182	85.09	2.14(1.84–2.47)
Hematologic malignancies	152	94.77	1.60(1.36–1.88)	3	1.26	2.39(0.49–6.97)	149	93.52	1.59(1.35–1.87)
All Others	117	45.26	2.58(2.14–3.10)	1	0.40	2.47(0.06–13.76)	116	44.86	2.59(2.14–3.10)

SIR Standardized incidence ratio; CI confidence interval; N/A not applicable.

### Predictors of second non-breast primary malignancies in breast cancer patients

Using the *SIR* method, we highlighted the increasing risk of developing a non-breast malignancy in breast cancer patients, as compared with the age- and gender-matched general population (Table 2 and Fig 1). We also used Cox proportional hazards analysis to investigate factors predicting higher SPM risk within breast cancer patients (Table 4). Notably, older age [increased age by every ten years, hazard ratio (HR): 1.45 (1.40–1.49); p < 0.0010], male gender [HR: 3.01; 95% CI: 2.38–3.80; p < 0.001], use of chemotherapy (HR: 1.26; 95% CI: 1.16–1.36; p < 0.001), and the presence of liver cirrhosis (HR: 2.84; 95% CI: 2.32–3.47; p < 0.001) were found to be significantly associated with the incidence of a second non-breast malignancy in breast cancer patients (Table 4).

**Table 4:** Risk factors for secondary primary cancers development in patients with breast cancer. Multivariable analysis: *p*<0.1 enter.

	Univariate analysis		Multivariateanalysis^a^	
Variables	HR (95% CI)	*P* Value	HR (95% CI)	*P* Value
Age increases per 10 years	1.46(1.42–1.50)	<0.001	1.45(1.40–1.49)	<0.001
Gender (male)	4.54(3.60–5.72)	<0.001	3.01(2.38–3.80)	<0.001
**Comorbidities**				
Diabetes mellitus	1.60(1.46–1.75)	<0.001	1.06(0.96–1.17)	0.265
COPD	1.46(1.33–1.61)	<0.001	1.05(0.95–1.16)	0.379
Chronic kidney disease	1.48(1.31–1.68)	<0.001	1.11(0.97–1.27)	0.116
Liver cirrhosis	3.76(3.09–4.59)	<0.001	2.84(2.32–3.47)	0.000
Autoimmune diseases	1.39(1.22–1.59)	<0.001	1.12(0.98–1.28)	0.101
Dyslipidemia	1.55(1.43–1.69)	<0.001	1.08(0.99–1.19)	0.096
**Breast Cancer Treatment**				
Hormone treatment	0.98(0.90–1.06)	0.556		
Chemotherapy	0.94(0.87–1.01)	0.086	1.26(1.16–1.36)	<0.001
Trastuzumab	1.09(0.86–1.39)	0.463		
Radiotherapy	1.01(0.94–1.09)	0.812		

Abbreviation: COPD = Chronic obstructive pulmonary disease; ESRD = End-stage renal disease

^a^All factors with *p*< 0.1 in univariate analyses were included in the Cox multivariate analysis.

## Discussion

Development of a second primary malignancy is one of the most significant concerns for breast cancer survivors, especially in the current era where breast cancer survival has been significantly improved by new treatments and early detection.[9] Accordingly, a growing need exists for evaluating the risk of second primary malignancy in both male and female breast cancer patients. In the present study, 101,493 breast cancer patients, including 578 male patients and 100,915 female patients, were analyzed for a total of 529,782 patient-years. To the best of our knowledge, this is the largest population-based study to investigate the risk for developing a subsequent non-breast malignant disease in Asian breast cancer patients. Furthermore, the extensive coverage of the NHI, their strict diagnostic criteria, and the data available from the large and detailed database maintained by the NHIRD, facilitated an unbiased study of second non-breast malignancy incidence in breast cancer patients and eliminated potential confounding factors.

Several reports have suggested that differences in hormonal and genetic predispositions between male and female patients can affect breast cancer diagnoses and clinical outcomes.[4,14] In the present study, we showed that breast cancer survivors of both genders exhibited a significantly higher risk for developing another primary malignancy compared with the general population and that male breast cancer patients had a higher excess risk compared with female breast cancer patients. (Fig 1 and Table 2) While the risk of second primary malignancy in female breast cancer patients has been addressed in different cohorts[7–9,15], fewer studies have examined a potential increased risk in male breast cancer patients. Aside from our study, Curtis and his colleagues analyzed data from the Surveillance, Epidemiology, and End Result Program (SEER) database, which enrolled patients that were diagnosed during the year from 1970 to 2000, and showed a marginal increased risk of subsequent cancer in male breast cancer patients (ratio of observed to expected cancer = 1.11), whereas the increased risk in female patients was significant (O/E = 1.18).[9,16] Differences in the risk patterns presented by Curtis et al. compared with those in the current study may reflect the underlying racial or ethnic disparities, differences of life-style and environmental risks or changes in treatment. It is important to note that a higher percentage of male breast cancer patients had comorbid diseases, such as DM, COPD, CKD and liver cirrhosis, compared with female patients, whereas female patients were more likely to have autoimmune diseases at the time of breast cancer diagnosis. (Table 1) The underlying comorbidities may also impact the overall risk of developing second primary cancer.

In the present study, we show that the age at breast cancer diagnosis was a major determinant of the relative risk for subsequent malignancy in breast cancer patients of both genders. The indicated SIRs for patients diagnosed with breast cancer at an age of 30 years or younger, was more than five times higher than that of patients who were diagnosed at an age greater than 80 years. These findings are consistent with previously published data.[7,9,11] Some researchers have argued that the increased risk for a second cancer in younger patients may be related to the administration of intensive treatments and/or a longer follow-up period.[17–20] However, in the present study and in studies using Scandinavian cohort data[7], age-related risk differences remained significant even after the adjustment for possible confounding factors, such as calendar year of breast cancer diagnosis, duration of follow-up, and breast cancer treatment. These findings suggest that certain genetic predispositions are associated with early onset breast cancer and may contribute to the higher risk of developing a second primary cancer[21]. The higher SIR we observed in young breast cancer patients may reflect this distinct biology presented in young breast cancer patients and a relative lower cancer incidence in the age-matched general cohort.

When specific types of cancer were assessed, male and female patients of the current cohort were found to have distinct patterns of involvement (Table 3 and Fig 2). These findings highlight several points. First, genetic alternations may contribute to the development of second malignancy in breast cancer patients. According to literatures, the mutations of BRCA genes were associated with increased risk of breast, ovary and colon cancer, whereas the elevated risk of uterine cancer, thyroid cancer and kidney cancer correlated with loss-of-function mutations in PTEN[21–23]. Though information regarding these genetic alterations were lacking, we observed increasing risk of above-mentioned cancers after breast cancer diagnosis in our patients, particularly an increase in SIRs for colon and thyroid cancers in male breast cancer patients and ovarian and kidney cancers in female patients. Second, treatments for breast cancer may affect the risk of developing a second malignancy. In line with previous reports[9,11], we found an increased risk of developing thyroid, lung, stomach, pancreas, skin, uterus, head and neck cancers, as well as hematologic malignancies in our cohorts. The developments of the cancers listed above were considered to be associated with radiotherapy, chemotherapy, and tamoxifen (for uterus cancer)[9,11]. In our study, we also analyzed the impacts of different treatment modalities in a multivariate analysis. For all cancer in general, only chemotherapy remained a significant factor after adjusting for other risk factors, including age, sex and comorbidities (Table 4). When we focused on uterus cancer, the impact of tamoxifen was shown (Table B in S1 File). Additionally, the relatively small number of cases in certain cancer types, especially in male breast cancer patients, may also limit the statistical power. Third, smoking and other life-style factors may influence the risk of second primary cancer [24,25]. Limited by our database, the exact smoking condition was unknown in our patients, but we found high prevalence of COPD in our cohort, especially in male patients (Table 1). Interestingly, if we assumed COPD as a surrogate for smoking exposure, we found that COPD was significantly associated with increasing risks of subsequent lung cancer (Table C in S1 File). Furthermore, in line with previous studies [9], a significant decrease in the risk of subsequent cervical cancer in female breast cancer patients was observed in the present study. The exact cause for the decreasing risk was not fully understood, but Curtis et al. had postulated that lower rate of smoking and lifestyle differences among breast cancer survivors in the SEER database may be responsible for this “protective”effect [9]. As mentioned above, we’re not able to investigate the impact of these personal habits to the risk of cervical cancer in our cohort, and further studies are needed. Lastly, frequent medical consultation and image examinations, such as computerized tomography scan of chest performed for staging or follow-up of breast cancer, may increase the detection of cancers, like thyroid, lung and colon cancer, and contribute to the higher SIRs we observed in present study.

In the present study, we also sought for possible risk factors of developing a non-breast primary malignancy in breast cancer patients. Similar to past reports[11], older patients and patients who had received chemotherapy were associated with a significantly higher risk of developing a second non-breast primary cancer (Table 4). The higher risk of developing a subsequent malignancy we shown in older breast cancer patients may be explained by the notion that aging is an important risk factor for most of the cancer types. In addition, we found that male gender and the presence of liver cirrhosis also significantly impacted this second cancer risk. Liver cirrhosis is a clear contributor to liver cancer and also to malignancies of the esophagus, pancreas and lung[26]. In this study, we found that cirrhosis significantly contribute to the risk of subsequent liver cancer in breast cancer patients (Table D in S1 File). In contrast, the rising risk of male gender observed in the multivariate analysis echoes the findings shown by SIR analysis, which may further suggest the inherited aggressive nature of male breast cancer.

There were several limitations associated with the present study. First, the possibility of synchronous cancer could not be completely excluded. However, most of the patients underwent complete imaging and laboratory surveys for breast cancer staging upon receiving a diagnosis. The patterns of the SIRs were similar for the general cohort and cohorts that excluded patients who were diagnosed with second primary cancers within the first year following their breast cancer diagnosis (Table 3 and Table A in S1 File), suggesting that the number of synchronous cancers was minimized. Second, due to a limitation of the NHIRD database, we were unable to access several important clinical factors, such as disease stage and specific molecular profiles, including expression of estrogen receptor, progesterone receptor, HER2 and other genetic alterations such as BRCA. In addition, factors such as obesity, smoking, alcohol use, and family history of malignancy were not included in the database. Finally, the relative short follow-up period and small number of male patients in present study may limit the scope of observation. As carcinogenesis can occur over an extended period of time, a longer follow-up period may be necessary to detect certain types of cancer. Further studies with larger patient cohort and longer observation are warranted.

In conclusion, the results from our nationwide population-based study demonstrate that breast cancer patients have an increased risk of developing non-breast primary malignant disease compared with the general population, and the excess risk of cancer was significantly higher for male breast cancer patients and/or patients who were diagnosed with breast cancer at a younger age. Furthermore, male gender, older age, previous chemotherapy treatment and comorbidity with liver cirrhosis were independent predictors of second primary malignancy in breast cancer patients. Based on the longer survival periods that are currently achieved for breast cancer patients, more intensive surveillance for a second primary cancer in all breast cancer patients, especially those patients exhibiting high-risk features, is needed to allow for earlier detection and treatment of second primary malignancies.

## Supporting Information

S1 File Supplementary tables. Table A. Standardized incidence ratios for specific cancer types among patients with breast cancer (including subsequent malignancy occurred within one year). Table B. Risk factors for uterine cancer development in patients with breast cancer. Table C. Risk factors for lung cancers development in patients with breast cancer. Table D. Risk factors for liver cancers development in patients with breast cancer.
(DOCX)
