# Supplementary material for: Risk of Second Non-Breast Primary Cancer in Male and Female Breast Cancer Patients: A Population-Based Cohort Study
Source: PLoS One. 2016 Feb 19;11(2):e0148597. doi: 10.1371/journal.pone.0148597 (PMC4760946; doi:10.1371/journal.pone.0148597)
Supplement: S1 File — Table A. Standardized incidence ratios for specific cancer types among patients with breast cancer (including subsequent malignancy occurred within one year). Table B. Risk factors for uterine cancer development in patients with breast cancer. Table C. Risk factors for lung cancers development in patients with breast cancer. Table D. Risk factors for liver cancers development in patients with breast cancer. (DOCX) [file pone.0148597.s001.docx]

**Table A. Standardized incidence ratios for specific cancer types among patients with breast cancer (including subsequent malignancy occurred within one year)**

|  | Total |  |  |  | Male |  |  |  | Female |  |  |
| --- | --- | --- | --- | --- | --- | --- | --- | --- | --- | --- | --- |
| Site of cancers | Observed | Expected | SIR (95% CI) |  | Observed | Expected | SIR (95% CI) |  | Observed | Expected | SIR (95% CI) |
| All cancers | 3,153 | 2,090.51 | 1.51(1.46–1.56) |  | 73 | 33.63 | 2.17(1.70–2.73) |  | 3,080 | 2,056.89 | 1.50(1.44–1.55) |
| Head and neck | 99 | 86.05 | 1.15(0.94–1.40) |  | 13 | 2.95 | 4.41(2.35–7.54) |  | 86 | 83.10 | 1.03(0.83–1.28) |
| Digestive | 1121 | 862.58 | 1.30(1.22–1.38) |  | 23 | 14.91 | 1.54(0.98–2.31) |  | 1,098 | 847.67 | 1.30(1.22–1.37) |
| Esophagus | 12 | 11.22 | 1.07(0.55–1.87) |  | 0 | 0.92 | 0.00(0.00–4.00) |  | 12 | 10.30 | 1.17(0.60–2.04) |
| Stomach | 151 | 110.49 | 1.37(1.16–1.60) |  | 4 | 2.39 | 1.67(0.46–4.28) |  | 147 | 108.10 | 1.36(1.15–1.60) |
| Colon and rectum, Anus | 555 | 403.65 | 1.37(1.26–1.49) |  | 12 | 5.51 | 2.18(1.13–3.80) |  | 543 | 398.15 | 1.36(1.25–1.48) |
| Liver and biliary tract | 340 | 289.65 | 1.17(1.05–1.31) |  | 6 | 5.41 | 1.11(0.41–2.41) |  | 334 | 284.24 | 1.18(1.05–1.31) |
| Pancreas | 63 | 47.56 | 1.32(1.02–1.69) |  | 1 | 0.68 | 1.48(0.04–8.24) |  | 62 | 46.89 | 1.32(1.01–1.70) |
| Lung and mediastinum | 426 | 279.31 | 1.53(1.38–1.68) |  | 11 | 5.85 | 1.88(0.94–3.36) |  | 415 | 273.46 | 1.52(1.38–1.67) |
| Bone and Soft tissue | 43 | 20.16 | 2.13(1.54–2.87) |  | 0 | 0.25 | 0.00(0.00–14.83) |  | 43 | 19.91 | 2.16(1.56–2.91) |
| Skin | 58 | 52.10 | 1.11(0.85–1.44) |  | 6 | 0.73 | 8.24(3.02–17.94) |  | 52 | 51.37 | 1.01(0.76–1.33) |
| Genitourinary | 849 | 521.60 | 1.63(1.52–1.74) |  | 12 | 6.79 | 1.77(0.91–3.09) |  | 837 | 514.81 | 1.63(1.52–1.74) |
| Cervix | 159 | 186.29 | 0.85(0.73–1.00) |  | N/A | N/A | N/A |  | 159 | 186.29 | 0.85(0.73–1.00) |
| Uterus | 298 | 111.94 | 2.66(2.37–2.98) |  | N/A | N/A | N/A |  | 298 | 111.94 | 2.66(2.37–2.98) |
| Ovary | 171 | 83.60 | 2.05(1.75–2.38) |  | N/A | N/A | N/A |  | 171 | 83.60 | 2.05(1.75–2.38) |
| Prostate | 8 | 4.26 | 1.88(0.81–3.70) |  | 8 | 4.26 | 1.88(0.81–3.70) |  | N/A | N/A | N/A |
| Bladder | 72 | 59.62 | 1.21(0.94–1.52) |  | 3 | 1.70 | 1.76(0.36–5.15) |  | 69 | 57.92 | 1.19(0.93–1.51) |
| Kidney | 141 | 75.88 | 1.86(1.56–2.19) |  | 1 | 0.83 | 1.21(0.03–6.74) |  | 140 | 75.06 | 1.87(1.57–2.20) |
| Thyroid | 238 | 102.56 | 2.32(2.04–2.64) |  | 2 | 0.15 | 13.20(1.60–47.69) |  | 236 | 102.40 | 2.30(2.02–2.62) |
| Hematologic malignancies | 192 | 112.06 | 1.71(1.48–1.97) |  | 4 | 1.51 | 2.66(0.72–6.80) |  | 188 | 110.56 | 1.70(1.47–1.96) |
| All Others | 127 | 54.10 | 2.35(1.96–2.79) |  | 2 | 0.49 | 4.06(0.49–14.67) |  | 125 | 53.61 | 2.33(1.94–2.78) |

SIR Standardized incidence ratio; CI confidence interval; N/A not applicable.

**Table B. Risk factors for uterine cancer development in patients with breast cancer**

**Multivariable analysis: *p*<0.1 enter**

|  | Univariate analysis | |  | Multivariateanalysis^a^ | |
| --- | --- | --- | --- | --- | --- |
| Variables | HR (95% CI) | *P* Value |  | HR (95% CI) | *P* Value |
| Age increases per 10 years | 1.28 (1.16–1.40) | < 0.001 |  | 1.26 (1.15–1.38) | < 0.001 |
| **Comorbidities** |  |  |  |  |  |
| Diabetes mellitus | 1.11 (0.80–1.54) | 0.530 |  |  |  |
| COPD | 1.13 (0.80–1.61) | 0.482 |  |  |  |
| Chronic kidney disease | 0.97 (0.59–1.61) | 0.908 |  |  |  |
| Liver cirrhosis | 0.41 (0.06–2.88) | 0.367 |  |  |  |
| Autoimmune diseases | 1.42 (0.92–2.20) | 0.115 |  |  |  |
| Dyslipidemia | 1.04 (0.76–1.42) | 0.809 |  |  |  |
| **Breast Cancer Treatment^b^** | |  |  |  |  |
| Hormone treatment |  |  |  |  |  |
| Anastrozole | 1.50 (1.08–2.09) | 0.016 |  | 1.29 (0.93–1.80) | 0.130 |
| Letrozole | 1.09 (0.72–1.65) | 0.675 |  |  |  |
| Exemestane | 0.55 (0.24–1.23) | 0.145 |  |  |  |
| Tamoxifen | 1.85 (1.38–2.49) | < 0.001 |  | 1.79 (1.33–2.40) | < 0.001 |
| Chemotherapy | 1.03 (0.81–1.31) | 0.833 |  |  |  |
| Trastuzumab | 0.87 (0.36–2.11) | 0.757 |  |  |  |
| Radiotherapy | 0.91 (0.71–1.16) | 0.438 |  |  |  |

Abbreviation: COPD = Chronic obstructive pulmonary disease; ESRD = End-stage renal disease

^a^All factors with *p*< 0.1 in univariate analyses were included in the Cox multivariate analysis.

^b^Treatment as time-dependent covariate in Cox model

**Table C. Risk factors for lung cancers development in patients with breast cancer**

**Multivariable analysis: *p*<0.1 enter**

|  | Univariate analysis | |  | Multivariateanalysis^a^ | |
| --- | --- | --- | --- | --- | --- |
| Variables | HR (95% CI) | *P* Value |  | HR (95% CI) | *P* Value |
| Age increases per 10 years | 1.54 (1.43–1.66) | < 0.001 |  | 1.50 (1.38–1.63) | < 0.001 |
| Gender (male) | 5.05 (2.77–9.18) | < 0.001 |  | 3.02 (1.64–5.54) | < 0.001 |
| **Comorbidities** |  |  |  |  |  |
| Diabetes mellitus | 1.43 (1.12–1.83) | 0.005 |  | 0.83 (0.63–1.09) | 0.185 |
| COPD | 1.96 (1.54–2.49) | < 0.001 |  | 1.37 (1.07–1.76) | 0.014 |
| Chronic kidney disease | 0.95 (0.63–1.44) | 0.824 |  |  |  |
| Liver cirrhosis | 1.61 (0.72–3.62) | 0.245 |  |  |  |
| Autoimmune diseases | 1.91 (1.39–2.61) | < 0.001 |  | 1.47 (1.07–2.03) | 0.018 |
| Dyslipidemia | 1.85 (1.49–2.30) | < 0.001 |  | 1.30 (1.02–1.65) | 0.037 |
| **Breast Cancer Treatment** | |  |  |  |  |
| Hormone treatment | 0.77 (0.63–0.95) | 0.014 |  | 0.72 (0.59–0.88) | 0.002 |
| Chemotherapy | 0.80 (0.66–0.97) | 0.025 |  | 1.09 (0.89–1.35) | 0.397 |
| Trastuzumab | 0.85 (0.40–1.79) | 0.662 |  |  |  |
| Radiotherapy | 1.04 (0.85–1.28) | 0.687 |  |  |  |

Abbreviation: COPD = Chronic obstructive pulmonary disease; ESRD = End-stage renal disease

^a^All factors with *p*< 0.1 in univariate analyses were included in the Cox multivariate analysis.

**Table D. Risk factors for liver cancers development in patients with breast cancer**

**Multivariable analysis: *p*<0.1 enter**

|  | Univariate analysis | |  | Multivariateanalysis^a^ | |
| --- | --- | --- | --- | --- | --- |
| Variables | HR (95% CI) | *P* Value |  | HR (95% CI) | *P* Value |
| Age increases per 10 years | 1.91 (1.76–2.07) | < 0.001 |  | 1.84 (1.67–2.03) | < 0.001 |
| Gender (male) | 3.47 (1.55–7.79) | 0.003 |  | 1.69 (0.75–3.81) | 0.207 |
| **Comorbidities** |  |  |  |  |  |
| Diabetes mellitus | 3.18 (2.52–4.01) | < 0.001 |  | 1.62 (1.24–2.10) | < 0.001 |
| COPD | 1.61 (1.20–2.15) | 0.001 |  | 0.79 (0.58–1.07) | 0.121 |
| Chronic kidney disease | 2.52 (1.83–3.47) | < 0.001 |  | 1.37 (0.98–1.92) | 0.068 |
| Liver cirrhosis | 25.27 (19.03–33.55) | < 0.001 |  | 16.63 (12.38–22.35) | < 0.001 |
| Autoimmune diseases | 1.50 (1.01–2.23) | 0.045 |  | 0.96 (0.64–1.44) | 0.843 |
| Dyslipidemia | 1.97 (1.54–2.52) | < 0.001 |  | 0.92 (0.70–1.21) | 0.537 |
| **Breast Cancer Treatment** | |  |  |  |  |
| Hormone treatment | 1.02 (0.80–1.30) | 0.894 |  |  |  |
| Chemotherapy | 0.82 (0.66–1.02) | 0.075 |  | 1.50 (1.18–1.91) | 0.001 |
| Trastuzumab | 1.02 (0.48–2.16) | 0.953 |  |  |  |
| Radiotherapy | 0.67 (0.53–0.85) | 0.001 |  | 0.87 (0.68–1.11) | 0.267 |

Abbreviation: COPD = Chronic obstructive pulmonary disease; ESRD = End-stage renal disease

^a^All factors with *p*< 0.1 in univariate analyses were included in the Cox multivariate analysis.
